# Multi-Approach Assessment for Stress Evaluation in Rainbow Trout Females, *Oncorhynchus mykiss* (Walbaum, 1792) from Three Different Farms during the Summer Season

**DOI:** 10.3390/ani11061810

**Published:** 2021-06-17

**Authors:** Paul Uiuiu, Călin Lațiu, Tudor Păpuc, Cristina Craioveanu, Andrada Ihuț, Alexandru Sava, Camelia Răducu, Cosmin Șonea, Radu Constantinescu, Daniel Cocan, Vioara Mireșan

**Affiliations:** 1 Department of Fundamental Sciences, Faculty of Animal Science and Biotechnologies, University of Agricultural Sciences and Veterinary Medicine Cluj-Napoca, 3-5 Mănăştur Street, RO-400372 Cluj-Napoca, Romania; paul.uiuiu@usamvcluj.ro (P.U.); calin.latiu@usamvcluj.ro (C.L.); ptudor2008@yahoo.com (T.P.); ihut.andrada@usamvcluj.ro (A.I.); alexsava_bv@yahoo.ro (A.S.); camelia.raducu@usamvcluj.ro (C.R.); vioara.miresan@usamvcluj.ro (V.M.); 2 Sociobiology and Insect Ecology Lab, Department of Taxonomy and Ecology, Faculty of Biology and Geology, Babes-Bolyai University, 44 Gheorghe Bilaşcu Street, RO-400015 Cluj-Napoca, Romania; cristinacraioveanu@gmail.com; 3 Department of Animal Production and Public Health, Faculty of Veterinary Medicine, University of Agricultural Sciences and Veterinary Medicine Bucharest, Splaiul Independenței nr.105, Sector 5, RO-050097 Bucharest, Romania; cosmin_sn@yahoo.com

**Keywords:** erythrocyte indices, hormones, PCA, rainbow trout welfare, temperature

## Abstract

**Simple Summary:**

Rainbow trout is one of the main freshwater fish species farmed in Romania, in a temperate continental climate. Blood biochemistry parameter values are well known for humans and other higher vertebrates, including some species of fish, and are fundamental for the assessment of physiological status. In this study, we examined the physicochemical parameters of water quality and the blood profile (hematological and hormonal profiles, antioxidant enzyme activities) of rainbow trout females reared in three farms, during the summer season, to monitor stress. These findings permit the progress of knowledge of blood parameters in fish and will encourage efforts to expand hematological and biochemical studies to assess the health of salmonids, which is essential for the sustainable development of aquaculture.

**Abstract:**

Blood biochemistry parameters are valuable tools for monitoring fish health. Their baseline values are still undefined for a multitude of farmed fish species. In this study, changes in the blood profile of rainbow trout females (*Oncorhynchus mykiss*) from three farms were investigated using different biomarkers during the summer season. In the given context, the main water physicochemical parameters were investigated and twelve biochemical parameters were measured from blood samples of rainbow trout reared in the Fiad, Șoimul de Jos, and Strâmba farms. We selected these farms because the genetic background of the rainbow trout is the same, with all studied specimens coming from the Fiad farm, which has an incubation station. Forty-five samples were collected monthly (May to August) throughout summer to observe the changes in the blood profile of rainbow trout. Principal component analysis showed a clear separation both among the studied farms and months. Furthermore, significant correlations (*p* < 0.05) between the majority of the biochemical parameters were found, indicating that the environmental parameters can influence several blood parameters at the same time. The present study provides several useful norms for assessing the welfare of rainbow trout, indicating that the relationships among different parameters are important factors in interpreting the blood biochemical profiles.

## 1. Introduction

Rainbow trout *Oncorhynchus mykiss,* Walbaum, 1792, is the main target of Romanian aquaculture. The total aquaculture production in Romania in 2019 was 12,847 tons [1], and rainbow trout production in 2017 was 1840 tons [2]. However, rainbow trout production is higher in farms, but official reporting is not conducted properly. Compared to wild rainbow trout, farmed fish are exposed to intensive rearing conditions, which can affect welfare and growth [3]. Knowledge regarding the physiological changes of fish, as a response to stressors, can improve productivity performances, fish welfare, and product quality [3,4]. Harvesting-related stimuli, such as netting, loading, and unloading, are usually unavoidable and cause detectable physiological responses in food fish that can be deleterious to product quality and value. The struggle of fish during harvest has been shown to result in rapid decreases in muscle pH post-mortem, affecting fillet quality through loss of fillet firmness and increased fillet gaping [5]. Environmental parameters can negatively influence the growth and health of salmonids by inducing a state of stress if their tolerances are exceeded. In humans, as well as in animals, stress can be considered a precursor of disease conditions [6]. Different methods can be used in ascertaining incipient forms of stress, such as hematological profiles [7,8], antioxidant enzymes [9,10,11], or hormonal level changes [6].

The initial physiological response of fish to stress involves the release of catecholamines and the activation of the hypothalamic–pituitary–interrenal axis (HPI) [12]. The stress response implies a series of mechanisms that facilitate the homeostasis control of an organism when it has been previously modified by the action of an intrinsic or extrinsic factor [13,14]. The main corticosteroid released by fish when facing different stressors is cortisol (F). Stress quantification by measuring F levels is an important index for fish welfare research [15]. Fast alterations of plasma corticosteroid and catecholamine concentrations are considered initial responses to common stress conditions [6]. Extensive literature is available about fish biology of stress and physiological and behavioral responses to a wide variety of physical, chemical, and biological stressors, both in wild and captivity conditions. Recent studies suggest that chronic and acute stress can negatively affect reproductive processes, metabolism, and production performances of fish [16,17,18,19,20,21]. 

Steroid hormones are involved in the regulation and progress of some physiological processes in vertebrates, such as embryonic development, sex differentiation, metabolism, immunological responses [13,22], circadian rhythms, stress responses, and reproduction [23]. The hypothalamus–pituitary–interrenal and the hypothalamus–pituitary–gonadal axes control steroid biosynthesis in teleost fishes, which usually occurs in peripheral tissues, such as the gonads, interrenal gland, and brain [23,24]. Fish gonads produce a series of estrogen, androgen, and progesterone steroids. Similarly to mammals, the main estrogen produced by female individuals of teleost fish species is 17β-estradiol [25]. Testosterone is also one of the most common secreta of teleost fish. However, in teleost fish, all these steroids can be present in the blood plasma of both females and males, as compared to mammals [13]. 

Most studies on this subject focus on relations among intrinsic (e.g., size, sex, physiological condition) and extrinsic factors (e.g., environmental conditions, handling, and feeding). The correlations between these factors and rainbow trout welfare represent an important and current topic [15,26,27,28]. These studies highlight the necessity of understanding the mechanisms responsible for inducing stress on physiological processes in farmed fish, especially the role of cortisol in adaptability to stress.

In this study, we evaluated the fluctuations of the hematologic and hormonal profiles, as well as oxidative stress during the summer season, in three fish farms, when rainbow trout are subjected to technological stressors (handling, transportation, high densities, feeding, etc.) and environmental stressors (high water temperature, low levels of dissolved oxygen, rainfall, river deposits, decomposing organic matter, dissolved solids, etc.).

## 2. Materials and Methods

### 2.1. Ethics

All procedures involving animals were conducted following Romanian (Law 43/2014) and European legislation (EU Directive 63/2010). The study was approved by the Ethics Committee of the University of Agricultural Sciences and Veterinary Medicine Cluj-Napoca (No. 148/2019).

### 2.2. Rainbow Trout Farms Description and Physicochemical Water Parameters

The study was carried out in three rainbow trout farms, in north-western Romania, in Bistrița-Năsăud county, namely Fiad (47°29′16.5″ N 24°22′59.4″ E), Strâmba (47°15′37.3″ N 24°40′10.2″ E) and Șoimul de Jos (47°09′55.8” N 24°50′03.2″ E) farms. These locations were selected because the rainbow trout genetic line found in all three farms are identical, with all studied individuals originating from Fiad farm, which has an incubation station. The feeding technique and the administered feed, Aller Gold (Aller Aqua, Christiansfeld, Denmark), were the same during the entire experimental period, with the chemical composition as presented in Table 1. The feeding rate (according to the suggested practices given by the producer of the food, 2% of biomass [27]) was identical for all fish in the same weight category during the study (251.39 ± 11.83 g) in all three farms. Noise levels were not determined due to the lack of anthropogenic activities (agricultural, forestry, and transportation) in the nearby area. Only ambient and natural noises were noted. Stocking densities in all farms were similar, between 15 and 20 kg/m^3^.

The recorded physicochemical parameters of water were: temperature (°C), dissolved oxygen (mg/L), pH (pH/ORP meter), total dissolved solids (TDSs), conductivity (µS/cm), resistivity (Ω/cm), salinity (PSU), ammonia (mg/L), nitrites (mg/dm^3^) and nitrates (mg/dm^3^). Temperature, dissolved oxygen, pH, TDS, conductivity, resistivity, and salinity were monitored at sunrise, at 8 A.M., before feeding, at different points of the ponds (inlet, center, and outlet), using the Hanna HI 9828/4-01 multiparameter. The methods used for determining ammonia (NH_3_), STAS 9800-2/71, nitrites (NO_2_), SR ISO 6777/1996, and nitrates (NO_3_), SR ISO 7890:1-1998, were applied by spectrometry. These parameters were monitored monthly, water samples being collected, transported, and analyzed at the Aquaculture Hygiene Laboratory of U.A.S.V.M. Cluj-Napoca, in less than four hours since collection, using a 0.5 L polypropylene container with a screw cap. 

### 2.3. Fish Sampling

Forty-five randomly selected fish (15 from each farm) were sampled every summer month (May, June, July, and August) of 2019. During the experimental period, a total of 180 rainbow trout (*Oncorhynchus mykiss*) females were selected. Fish were fasted 24 h before sampling. The individuals were removed from the ponds and were immediately anesthetized with clove oil (eugenol 30 mg L^−1^) to reduce handling stress [30]. Blood samples were collected during the morning (8–9 A.M.) by caudal vein puncture with a needle syringe (5 mL) and transferred in 5 mL blood sampling tubes with anticoagulant (Li-heparin) and serum separator tube, SST. After blood sampling, weighing, and photographic documentation, fish were transferred to the quarantine pond, being monitored for facilitating faster recovery from anesthesia.

### 2.4. Hematological and Antioxidant Enzyme Analysis

The hematological profile includes the red blood cell (RBC) count, hemoglobin (Hb), and hematocrit (Hct) from whole blood. The erythrocyte indices, mean corpuscular volume (MCV), mean corpuscular hemoglobin (MCH), and mean corpuscular hemoglobin concentration (MCHC) were calculated according to standard formulas [31]. For analyzing oxidative stress, the levels of two antioxidant enzyme levels were determined: superoxide dismutase (SOD) and glutathione peroxidase (GPx), according to established protocols [32,33,34,35]. The analyses were carried out using a Screen Master Touch UV–VIS spectrophotometer (Hospitex Diagnostics, Italy), in 340–620 nm wavelength interval. The utilized reagents were purchased from Hospitex Diagnostics, Florence, Italy. 

### 2.5. Hormonal Serum Profile Analysis (Serum Parameters)

Serum separator tubes (SSTs) were used for the chemical analysis of the blood. The samples were kept at 4 °C overnight, and the serum was obtained by centrifugation (15 min at 1000 xg). It was visually inspected to exclude hemolysis, which could influence results, and was stored at −80 °C until later analysis. The analyses were carried out using a HiPo MPP-96 Microplate Photometer spectrophotometer (BioSan, Rīga, Latvia) at 340–620 nm wavelength intervals. Plasma cortisol was quantified by ELISA, using a commercial kit from DiaMetra S.r.l. (Cortisol Ref. DKO001, Milano, Italy), according to the manufacturer’s prospect. The minimum detectable concentration is 2.42 ng/mL at a 95% confidence limit.

Testosterone (T), 17β-estradiol (E_2_), and progesterone (PROG) concentration in the serum were measured using the Fish Testosterone (T) Elisa, Fish 17β-estradiol (E_2_) Elisa, and Fish Progesterone (PROG) Elisa kits, according to manufacturer instructions. All Elisa kits were purchased from Aviva Systems Biology Co., Ltd. (San Diego, CA, USA). The minimum detectable concentration was 0.1 ng/mL for T, 40 pg/mL for E_2_ and 0.5 ng/mL for PROG. The variation coefficients for intra- and inter-tests were <10%.

### 2.6. Statistical Methods

To check whether data regarding stress response is associated with other parameters measured, with the location and/or with the month of measurement, we performed a preliminary exploratory analysis using the Principal Component Analysis (PCA) on the dataset of physiological parameters (stress response variables, hormones, hematological parameters). The physiological response variables with the strongest associations with the first two PC axes were further analyzed utilizing analyses of variance (either parametric or non-parametric, according to the distribution of the respective parameters), to check whether the location and/or month play important roles for the distribution of these parameters. We checked whether the data were normally distributed with the help of a Shapiro–Wilk test (shapiro.test function in RStudio). The following parameters were normally distributed: SOD and MCV. The values of GPx, cortisol, estradiol, testosterone, Ery, Hct, and MCH were not normally distributed. In the case of normally distributed data, we performed a two-way ANOVA followed by TukeyHSD post hoc test. For other data, we used the Kruskal–Wallis test followed by a pair-wise Wilcoxon post hoc test with Bonferroni correction. 

Next, as months tended to have a far stronger effect on the data, we performed correlations between monthly stress response variables and all other parameters (both environmental and physiological). We considered strong positive or negative correlations when the coefficient was >0.5 or <−0.5, and moderate positive or negative correlations when the coefficient was >0.3 and <=0.5, <−0.3 and <=−0.5, respectively (Supplementary Tables S1–S4). Values were considered significant if *p* < 0.05, and those *p*-values smaller than 0.001 were presented as *p* < 0.001. All analyses were performed in RStudio Version 1.2.5042 (2020). 

## 3. Results

**Figure 1:**
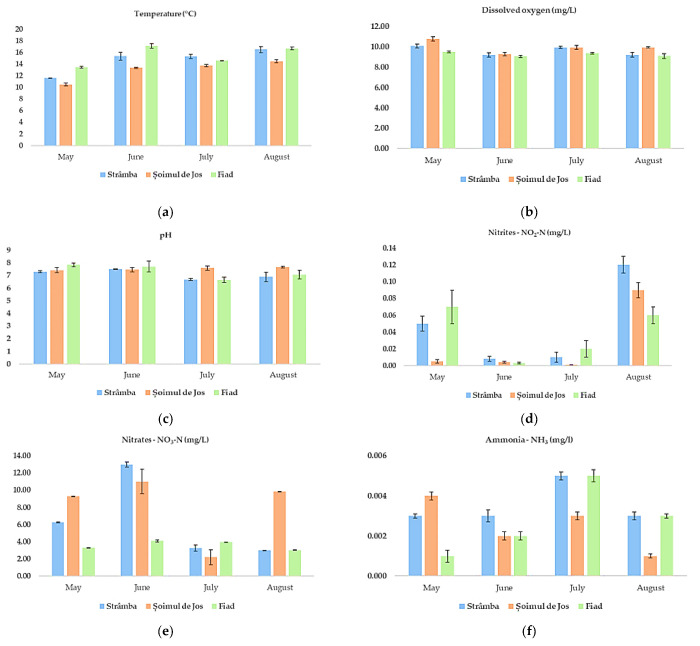

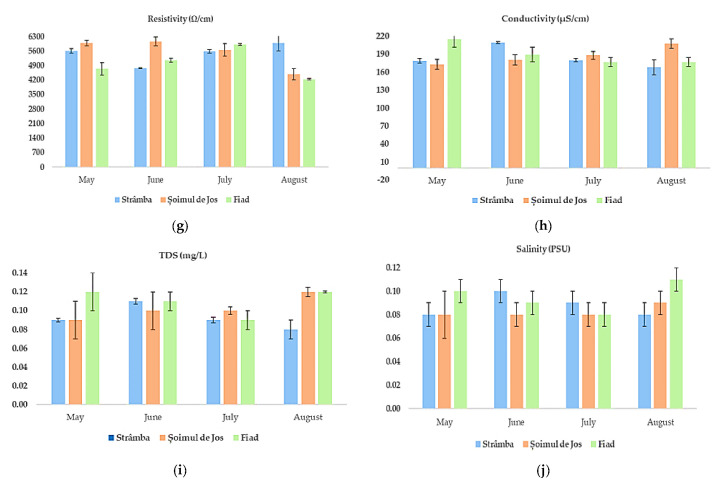
Physicochemical parameters of water (mean ± standard deviation) determined monthly during the summer season: (**a**) temperature (°C); (**b**) dissolved oxygen (mg/L); (**c**) pH (pH/ORP meter); (**d**); nitrites (mg/L) (**e**) nitrates (mg/L); (**f**) ammonia (mg/L); (**g**) resistivity (Ω/cm); (**h**) conductivity (µS/cm); (**i**) total dissolved solids (TDS); (**j**) salinity (PSU).

The results of the physicochemical water quality parameters measured in farms during the summer season are reported in Figure 1a–j. The physicochemical water parameters measured (at inlets, the center of ponds, and outlets) were averaged for each farm and month. Between farms, we found no significant differences in these parameters (one-way ANOVA for all parameters between farms had *p* > 0.05).

Regarding water temperature (°C) (Figure 1a), we observed that Fiad farm recorded the highest values, followed by Strâmba and Șoimul de Jos farms. Even though the mean determined water temperature was within the optimum limits for rainbow trout production, we observed an increase in temperature during the day, reaching even 21–22 °C in the afternoon. The dissolved oxygen-DO (mg/L) (Figure 1b), due to the strong negative correlation between temperature and DO, had the lowest levels in Fiad and the highest in Strâmba and Șoimul de Jos farms. 

The pH parameter (Figure 1c) did not show significant variation among months and farms, remaining approximately neutral during the summer season. The obtained values were always in accordance with rainbow trout requirements. This indicates that there is a very good capacity for water buffering in the farms and that the value fluctuations were within the normal limits of the rainbow trout life cycle. 

Regarding the nitrogen cycle in water ecosystems, the NO_2_ (Figure 1d), NO_3_ (Figure 1e) and NH_3_ (Figure 1f) parameters did not exceed the maximum permitted values for rainbow trout. Resistivity (Ω/cm), conductivity (µS/cm), total dissolved solids (TDS), (mg/L) and salinity (PSU) are all closely related to each other because the ability of water to resist or conduct an electric current is directly related to the amount of ionic material (salts) dissolved in the water. These conductive ions come from dissolved salts and inorganic materials such as alkalis, chlorides, sulfides, and carbonate compounds [36]. 

The Principal Component Analysis (PCA) revealed that the first (PC1) and the second (PC2) components explained 45.97% (23.93% and 22.04%, respectively) of the total variation (Table 2). Usually, PCA is applied to reduce a large number of variables into derived variables that can be easily visualized in 2- or 3-dimensional space. In our study, we used this technique to explore the correlation relationships that might exist between physiological parameters of rainbow trout in different farms and months. We used the loadings resulted from this analysis to find out which variables contribute most to the differences between female rainbow trout.

**Figure 2:**
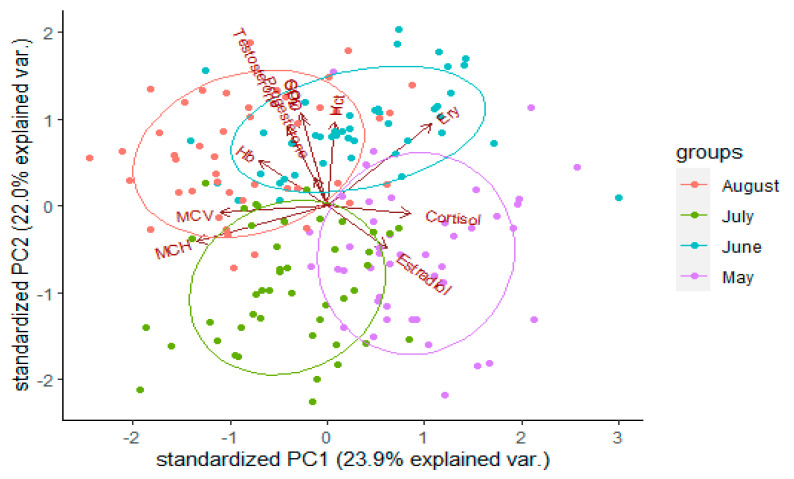
PCA of physiological parameters of rainbow trout (*Oncorhynchus mykiss*) measured in 2019 in four months, in three farms in Romania.

The strongest positive correlations with the PC1 axis were found in the following parameters: RBC, F, and E_2_. The parameters that correlated most strongly negatively correlated with the first axis were: MCH and MCV. The strongest positive correlations with the PC2 axis were found in the parameters: GPx, SOD, Hct, and T. The parameter most strongly negatively correlated with the second axis was E_2_. These parameters were not particularly associated with any farm; however, there was a clearer association between them and months (Figure 2). RBC was associated with June, F and E_2_ were associated with May, MCH and MCV were associated with July and August, and GPx, SOD, T, and Hct were associated with August and June (Figure 2).

**Figure 3:**
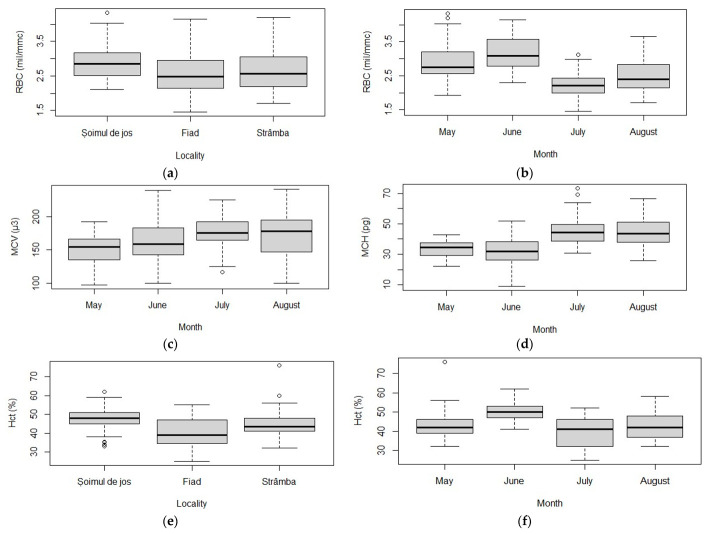
Hematological profile of rainbow trout (*Oncorhynchus mykiss*): (**a**) RBC counts measured in all months in different farms; (**b**) RBC counts measured in all farms in the different months; (**c**) MCV values measured in all farms in different months; (**d**) MCH values measured in all farms in different months; (**e**) Hct values measured in all months in the different farms; (**f**) Hct values measured in all farms in different months. Plots represent median (line inside boxes) values, 25–75 percent quartiles (boxes), and minimal and maximal values, shown with short horizontal lines (“whiskers”). Outliers are represented with an open circle sign.

RBC count varied significantly between farms (chi-squared = 12.193, df = 2, *p* = 0.002) (Table 3). Higher values of the RBC count were found in fish from Șoimul de Jos (2.85 × 1012/L) compared to fish from the other two farms (Fiad—2.48; Strâmba—2.57 × 1012/L) (Pairwise Wilcoxon rank sum test: *p* < 0.05 in all comparisons between Șoimul de Jos and Fiad, and Șoimul de Jos and Strâmba) (Figure 3a).

Similarly, the RBC count varied significantly between months (chi-squared = 66.388, df = 2, *p* < 0.001) (Table 3). RBC counts in May and June were higher than those measured in July and August (Pairwise Wilcoxon rank sum test: *p*_May–June_ = 0.106, *p*_May–July_ < 0.001, *p*_May–August_ = 0.003, *p*_June–July_ < 0.001, *p*_June–August_ < 0.001, *p*_July–August_ = 0.117) (Figure 3b). 

In May, the RBC count was strongly positively correlated with resistivity (rho = 0.67, *p* < 0.005) and DO (rho = 0.67, *p* < 0.005), and strongly negatively correlated with MCV (rho = −0.58, *p* < 0.005), temperature (rho = −0.67, *p* < 0.005), conductivity (rho = −0.67, *p* < 0.005), TDS (rho = −0.67, *p* < 0.005) and NO_2_ (rho = −0.67, *p* < 0.005). In June, the RBC count was strongly negatively correlated with MCV (rho = −0.91, *p* < 0.005) and MCH (rho = −0.79, *p* < 0.005). In July, the RBC count was strongly positively correlated with Hct (rho = 0.69, *p* < 0.005), F (rho = 0.56, *p* < 0.005), pH (rho = 0.65, *p* < 0.005), conductivity (rho = 0.65, *p* < 0.005), TDS (rho = 0.65, *p* < 0.005), DO (rho = 0.65, *p* < 0.005), and strongly negatively correlated with MCH (rho = −0.52, *p* = 0.003), MCHC (rho = −0.53, *p* = 0.002), NO_2_ (rho = −0.65, *p* < 0.001) and NO_3_ (rho = −0.65, *p* < 0.001). In August, the RBC count was strongly negatively correlated with MCV (rho = −0.7, *p* < 0.001) and MCH (rho = −0.65, *p* < 0.001).

The mean corpuscular volume (MCV) values varied significantly between months (F = 7.45, df = 3, *p* < 0.001) (Table 3). MCV values in May were lower than those in July and August (TukeyHSD test: *p*_May–July_ < 0.001, *p*_May–August_ = 0.002) (Figure 3c). In May, MCV values were strongly positively correlated with MCH (r = 0.67, *p* < 0.001), and strongly negatively correlated with RBC (r = −0.51, *p* = 0.0003). In June, MCV values were strongly positively correlated with Hct (r = 0.68, *p* < 0.001) and MCH (r = 0.76, *p* < 0.001), and strongly negatively correlated with RBC (r = −0.88, *p* < 0.001). In July, MCV values were strongly positively correlated with Hct (r = 0.6, *p* < 0.001) and strongly negatively correlated with NO_3_ (r = −0.52, *p* = 0.0003). In August, MCV values were strongly positively correlated with Hct (r = 0.56, *p* < 0.001) and strongly negatively correlated with RBC (r = −0.68, *p* < 0.001).

The mean corpuscular hemoglobin (MCH) values varied significantly between months (chi-squared = 62.88, df = 3, *p* < 0.001) (Table 3). MCH values in May and June were lower than those in July and August (Pairwise Wilcoxon rank sum test: *p*_May–July_ < 0.001, *p*_May–August_ < 0.001, *p*_June–July_ < 0.001, *p*_June–August_ < 0.001) (Figure 3d). In May, MCH values were strongly positively correlated with Hb (rho = 0.56, *p* < 0.001) and MCV (rho = 0.68, *p* < 0.001). In June, MCH values were strongly positively correlated with Hb (rho = 0.68, *p* < 0.001), MCV (rho = 0.78, *p* < 0.001), resistivity (rho = 0.59, *p* < 0.001), DO (rho = 0.56, *p* < 0.001), and strongly negatively correlated with RBC (rho = −0.79, *p* < 0.001), pH (rho = −0.56, *p* < 0.001), temperature (rho = −0.56, *p* < 0.001), conductivity (rho = −0.59, *p* < 0.001), TDS (rho = −0.59, *p* < 0.001), salinity (rho = −0.59, *p* < 0.001) and NO_3_ (rho = −0.56, *p* < 0.001). In July, MCH values were strongly positively correlated with Hb (rho = 0.57, *p* < 0.001) and MCHC (rho = 0.56, *p* < 0.001), and strongly negatively correlated with RBC (rho = −0.52, *p* < 0.001). In August, MCH values were strongly positively correlated with Hb (rho = 0.51, *p* = 0.0003), temperature (rho = 0.59, *p* < 0.001), NO_3_ (rho = 0.58, *p* < 0.001), and strongly negatively correlated with RBC (rho = −0.65, *p* < 0.001) and DO (rho = −0.59, *p* < 0.001).

The hematocrit (Hct) values varied significantly between locations (chi-squared = 30.673, df = 2, *p* < 0.001) and months (chi-squared = 46.23, df = 3, *p* < 0.001) (Table 3). The Hct values in Șoimul de Jos were significantly higher than those in the other two locations (Wilcoxon rank sum test: *p*_Șoimul de Jos-Fiad_ < 0.001, *p*_Șoimul de Jos-Strâmba_ = 0.001), and Hct values were higher in Strâmba than those measured in Fiad (*p*_Strâmba-Fiad_ = 0.01) (Figure 3e). 

The Hct values in June were significantly higher than those recorded in the other three months (Wilcoxon rank sum test: *p*_May–June_ < 0.001, *p*_June–July_ < 0.001, *p*_June–August_ < 0.001) (Figure 3f). In May, Hct values were strongly positively correlated with Hb (rho = 0.55, *p* < 0.001) and strongly negatively correlated with pH (rho = −0.58, *p* < 0.001), salinity (rho = −0.58, *p* < 0.001) and NH_3_ (rho = −0.58, *p* < 0.001). In June, Hct values were strongly positively correlated with MCV (rho = 0.58, *p* < 0.001). In July, Hct values were strongly positively correlated with RBC (rho = 0.69, *p* < 0.001), MCV (rho = 0.56, *p* < 0.001), T (rho = 0.57, *p* < 0.001), F (rho = 0.72, *p* < 0.001), pH (rho = 0.9, *p* < 0.001), conductivity (rho = 0.9, *p* < 0.001), TDS (rho = 0.9, *p* < 0.001) and DO (rho = 0.9, *p* < 0.001), and strongly negatively correlated with MCHC (rho = −0.72, *p* < 0.001), NO_2_ (rho = −0.9, *p* < 0.001) and NO_3_ (rho = −0.9, *p* < 0.001). In August, Hct values were strongly positively correlated with MCV (rho = 0.52, *p* = 0.0002) and strongly negatively correlated with MCHC (rho = −0.64, *p* < 0.001).

**Figure 4:**
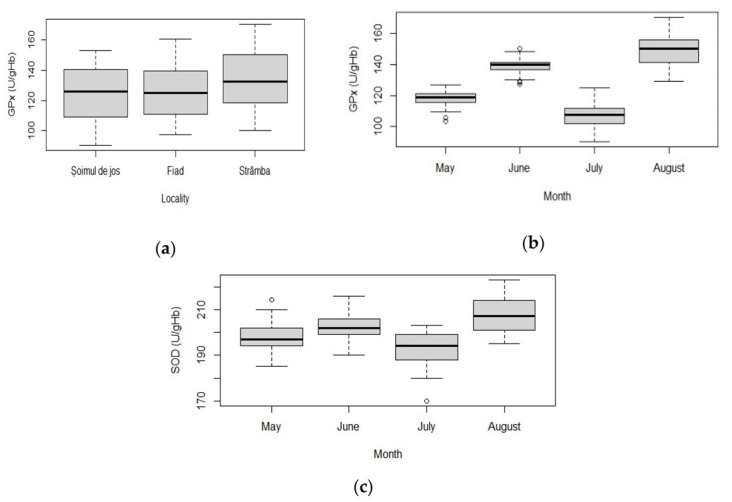
Antioxidant enzymes of rainbow trout (*Oncorhynchus mykiss*): (**a**) GPx values measured in all months in different farms; (**b**) GPx values in all farms in different months; (**c**) SOD values in all farms in different months. Plots represent median (line inside boxes) values, 25–75 percent quartiles (boxes), and minimal and maximal values, shown with short horizontal lines (“whiskers”). Outliers are represented with an open circle sign.

Glutathione peroxidase (GPx) of rainbow trout varied significantly between farms (chi-squared = 7.79, df = 2, *p* = 0.020) (Table 3). The Strâmba and Fiad farms were the most different (Pairwise Wilcoxon rank sum test: p_Strâmba-Fiad_ = 0.047) (Figure 4a). GPx values were significantly different in each month (chi-squared = 150.15, df = 3, *p* < 0.001; post hoc Pairwise Wilcoxon rank sum test: *p* < 0.001 in all comparisons) (Figure 4b). In June, GPx was strongly positively correlated with conductivity (rho = 0.6, *p* < 0.001), total dissolved solids (TDS) (rho = 0.6, *p* < 0.001), and salinity (rho = 0.6, *p* < 0.001), and strongly negatively correlated with resistivity (rho = −0.6, *p* < 0.001). In August, GPx was strongly positively correlated with resistivity (rho = 0.62, *p* < 0.001), NO_2_ (rho = 0.59, *p* < 0.001) and NH_3_ (rho = 0.59, *p* < 0.001), and strongly negatively correlated with pH (rho = −0.59, *p* < 0.001), conductivity (rho = −0.62, *p* < 0.001), TDS (rho = −0.62, *p* < 0.001) and salinity (rho = 0.62, *p* < 0.001).

The values of superoxide dismutase (SOD) varied significantly between months (F = 40.27, df = 3, *p* < 0.001) (Table 3). The farm factor had no influence on this parameter (F = 0.45, df = 2, *p* = 0.641) (Table 3). SOD values in each month were significantly different from each other (post hoc TukeyHSD test: *p* < 0.05 in all comparisons) (Figure 4c). 

**Figure 5:**
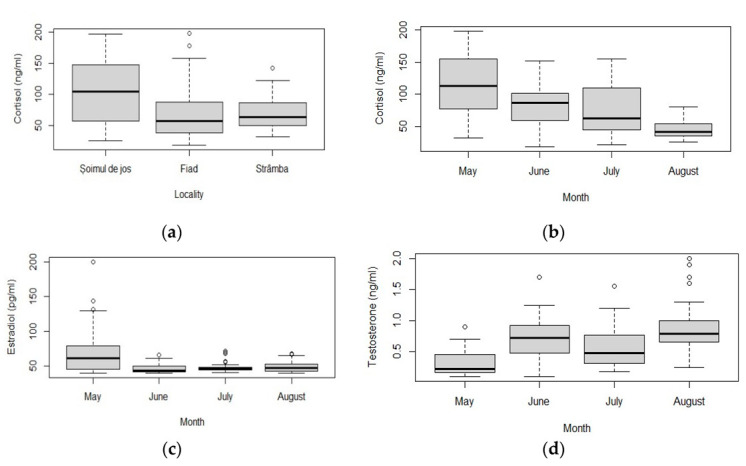
Hormonal profile of rainbow trout (*Oncorhynchus mykiss*): (**a**) F values measured in all months in different farms; (**b**) F values measured in all farms in different months; (**c**) E_2_ values measured in all farms in different months; (**d**) T values measured in all farms in different months. Plots represent median (line inside boxes) values, 25–75 percent quartiles (boxes), and minimal and maximal values, shown with short horizontal lines (“whiskers”). Outliers are represented with an open circle sign.

F varied significantly between farms (chi-squared = 19.269, df = 2, *p* < 0.001) (Table 3). Șoimul de Jos farm was the most different from the other two (Pairwise Wilcoxon rank sum test: *p*_Șoimul de Jos-Fiad_ < 0.001, *p*_Șoimul de Jos-Strâmba_ < 0.001) (Figure 5a.). F values were significantly different between months (chi-squared = 64.858, df = 3, *p* < 0.001) (Table 3). F levels were higher in May compared to the other three months (Pairwise Wilcoxon rank sum test: *p* < 0.005 in all comparisons). June had higher F levels than August, but lower than May (Pairwise Wilcoxon rank sum test: *p*_May–June_ = 0.004, *p*_June–August_ < 0.001); August had the lowest F levels compared to the other three months (Pairwise Wilcoxon rank sum test: *p* < 0.005 in all comparisons) (Figure 5b). In May, F was strongly positively correlated with resistivity (rho = 0.54, *p* < 0.001) and dissolved oxygen (rho = 0.54, *p* < 0.001), and strongly negatively correlated with temperature (rho = −0.54, *p* < 0.001), conductivity (rho = −0.54, *p* < 0.001), TDS (rho = −0.54, *p* < 0.001) and NO_2_ (rho = −0.54, *p* < 0.001). In July, F was strongly positively correlated with Hct (rho = 0.72, *p* < 0.001), RBC (rho = 0.56, *p* < 0.001), T (rho = 0.58, *p* < 0.001), pH (rho = 0.83, *p* < 0.001), conductivity (rho = 0.83, *p* < 0.001), TDS (rho = 0.83, *p* < 0.001) and dissolved oxygen (rho = 0.83, *p* < 0.001), and it was strongly negatively correlated with NO_2_ (rho = −0.83, *p* < 0.001), NO_3_ (rho = −0.83, *p* < 0.001), NH_3_ (rho = −0.58, *p* < 0.001) and temperature (rho = −0.58, *p* < 0.001).

E_2_ varied significantly between months (chi-squared = 24.618, df = 3, *p* < 0.001) (Table 3). May had the most different E_2_ values compared with the other three months (Pairwise Wilcoxon rank sum test: *p* < 0.05 in all comparisons) (Figure 5c). T varied significantly between months (chi-squared = 63.072, df = 3, *p* < 0.001) (Table 3). Significant differences were found between the values of T in May and those of the other months, and between July and August. (Pairwise Wilcoxon rank sum test: *p* < 0.05 in all comparisons) (Figure 5d). In May, T was strongly positively correlated with F (rho = 0.55, *p* < 0.001), resistivity (rho = 0.74, *p* < 0.001), DO (rho = 0.74, *p* < 0.001) and strongly negatively correlated with temperature (rho = −0.74, *p* < 0.001), conductivity (rho = −0.74, *p* < 0.001), TDS (rho = −0.74, *p* < 0.001) and NO_2_ (rho = −0.74, *p* < 0.001). In July, T was strongly positively correlated with Hct (rho = 0.57, *p* < 0.001), F (rho = 0.58, *p* < 0.001), conductivity (rho = 0.6, *p* < 0.001), TDS (rho = 0.6, *p* < 0.001) and DO (rho = 0.6, *p* < 0.001), and strongly negatively correlated with NO_2_ (rho = −0.6, *p* < 0.001) and NO_3_ (rho = −0.6, *p* < 0.001). The levels of E2 and T found circulating in the peripheral plasma of rainbow trout varied between 46.39 and 48.21 pg/mL for E2, and 0.44 and 0.61 ng/mL for T during the four months of the summer season.

The values of the physicochemical parameters are within normal limits for rainbow trout aquaculture, and the PCA showed that the differentiating parameters between months and farms are: RBC, F, E_2_, MCH, MCV, GPx, SOD, Hct, and T.

## 4. Discussion

The results obtained in this study highlight the fluctuations of the hematological and hormonal profiles, and the oxidative stress in rainbow trout from three Romanian farms during the summer season. In addition, we have established the main physiological and environmental parameters that significantly differentiate analyzed farms and months, to offer a baseline necessary in the understanding of their physiological role in the immunological system of rainbow trout. This baseline is specific to a temperate-continental climate, with four seasons, such as in Romania [37].

As other studies demonstrated, many biotic and abiotic factors can affect blood parameters in fish [22,38,39]. Seasonal differences in blood biomarkers may occur due to alterations in water physicochemical parameters, such as differences in temperature, dissolved oxygen concentration, rainfall, river deposits, decomposing organic matter, dissolved solids, etc. During summer, stress due to improper water temperatures and low dissolved oxygen levels is an increasingly common issue, severely affecting the growth performances of rainbow trout [6,22]. The data obtained in our study regarding the physicochemical parameters of the water in the three farms are in accordance with other studies [40,41,42,43,44], which follow the environmental parameters necessary for the normal development of rainbow trout. When allowed to behaviorally thermoregulate, rainbow trout have a mean preferred temperature of 16 °C but occupy a temperature range between 13 and 19 °C when oxygen is not limited [45].

Under the conditions of adequate values of dissolved oxygen in the water and through the nitrification process, the low amount of ammonia found in the studied farms can be transformed into nitrites and nitrates. Nitrates are less toxic to fish over a long period throughout the entire nitrification process [45]. Self-purification is performed well in conditions of sufficient amounts of dissolved oxygen, but in the case of low values of this parameter, correlated with high temperature, the degradations are more intense (multiple correlations) [45]. As a limitation, the ammonia levels cannot fully present the situation of studied months and farms. However, they do present the situation of the farms in the last week before sampling.

Changes in the red blood component with manifestations of low/high RBC, Hct, and Hb values are a key feature of the clinical picture in different fish species. Erythrocyte indices help in diagnosing and treating anemia for each species, but the values of these indices are affected by age, sex, body mass, population density, environmental and species parameters [46].

In our study, according to the PCA, the number of red blood cells (RBCs) varied significantly between farms. The analysis of the monthly variation of hematological parameters during the summer season showed that the number of RBCs was highest in May and June and lowest in July and August. As fish stockings, genetic background, feed, and age of fish were almost identical in the three farms, it is presumed that the differences in RBC count values occurred due to environmental parameters. Generally, the number of RBCs in the summer season is higher compared to that of other seasons, due to an intensified hematopoietic function caused by an increase in water temperature, a decrease in DO [47], an intensification of metabolism, or increased photoperiod [48]. In our study, temperature proved to be the main environmental factor that stimulates changes in RBC content. The values obtained by us fall within the standard limits of 1.55–4.45 × 10^12^/L [49]. 

The mean corpuscular volume (MCV) and the mean corpuscular hemoglobin (MCH) values varied significantly between months. In a study of hematological parameters, Lone et al. [50] recorded lower values of MCV (88 fL) compared to our study, for the same age group studied, due to a strong positive correlation with water temperature, a fact highlighted in our study. The MCH value is influenced by the stage of development of a fish (i.e., fry, juvenile, adult) and is also influenced by the water temperature. According to other studies, the normal values of MCH recorded are between 23.16 and 68.06 pg [49,51]. Since MCHCs were not significantly different between months and farms, there is nothing to suggest that there was a higher contribution of immature red blood cells in the groups. 

The hematocrit (Hct) values varied significantly between farms and months. Similar to RBC, the hematocrit is presumed to vary due to environmental parameters. As for the monthly variation, the Hct values in June were significantly higher than those recorded in the other three months. The reference limits of Hct are between 20% and 43% [49]. In general, metabolic changes, including intensifications of erythropoiesis, occur due to high water temperatures, low levels of DO, and increased photoperiod during the summer. [48]. In summer, rainbow trout activity is much lower compared to other seasons (autumn–winter), which could be responsible for the observed variation in Hct. 

The antioxidant defense system of the body, which contains enzymes such as superoxide dismutase (SOD), catalase (CAT), and glutathione peroxidase (GPx), is set to maintain the lowest potential levels of reactive oxygen species (ROS) in cells, and it is recognized as an essential component of an organism’s maintenance [52]. Previous studies have shown that changes in environmental parameters, such as flow rate, water temperature, DO, photoperiod, population density, manipulation, can disrupt the antioxidant balance, causing oxidative stress response and affecting the welfare and growth performance of fish [53,54,55,56].

In the current study, Glutathione peroxidase (GPx) activity varied significantly between farms (Strâmba and Fiad), higher values of GPx being recorded in Strâmba farm compared to the other two farms. This difference was most likely the result of higher temperature values and lower DO, the thermal stress being more noticeable. Both SOD and GPx activity varied significantly between months. In May and July, the SOD and GPx values were lower than in June and August, being strongly correlated with conductivity, temperature, DO, total dissolved solids, and strongly negatively correlated with resistivity. The recorded results of SOD and GPx indicate that the rainbow trout metabolic compromise was higher in periods with higher temperatures and lower oxygen concentrations characteristic of summer months. Higher levels of DO may help to improve the oxidative parameters to cope with hyperoxia stress and to alleviate damage to tissue cell membranes. Ritola et al. [56] reported that supersaturated DO can stimulate the SOD and CAT activities of rainbow trout. 

Blood cortisol (F) levels are initial indicators of acute and chronic stress in fish. The gills, intestines, and liver are important targets for F in fish. These organs reflect the two major actions of F in fish: regulation of hydromineral balance and energy metabolism. Other activities of F include reduced growth rate and suppression of reproductive and immune functions [57,58]. An increase in plasma F is the most widely used indicator of stress assessment in fish. Plasma F levels usually rise rapidly within minutes of exposure to acute stress. Returning to normal levels may take one or several hours. When the stressor is chronic, F levels may remain high, but below the maximum recorded levels [57]. In our study, F varied significantly between farms. F values after acute stress (netting and confinement) showed monthly variations and were higher in May compared to the other summer months. June had higher F levels than August, but lower than May. August had the lowest F levels compared to all analyzed months. This could have occurred due to the geographical positioning of the farms (spatial variations of the farms) and environmental parameters. Between months, the PCA of the physiological variables supported the values of the recorded stress, when the temperature was higher and the DO was lower. This suggests that acute stress may have deeper effects on fish at higher temperatures. Higher F levels in early summer (May–June) compared to late summer (July–August) suggested that there was either some attenuation of the stress response due to chronic exposure to improper temperatures (diurnal changes) and low oxygen, or that other mitigating factors, such as changes in water supply (water source), have led to different monthly responses. The values obtained in this study fall within the limits cited by Martínez-Porchas et al. [59], who state the following plasmatic F values from the literature review, before and after different stressors: *O. mykiss* after chemical exposure (49–110 nmol/L), handling and confinement (77–698 nmol/L), trapping (16–380 nmol/L, males; 57–764 nmol/L, females).

In teleost fish, testosterone (T), 11-ketotestosterone (11KT), progesterone (PROG), and estradiol-17b (E_2_) play an important role in hormonal control of reproductive functions. The main physiological function of steroid hormones in vertebrates is to regulate sexual development and reproduction. However, they have pleiotropic functions and beyond the “classical” function of the reproductive axis, they target several other physiological systems, including the immune system [60]. Several authors have reported impairment of growth and reproduction in fish due to environmental stressors, such as acid waters, pollutants, and resource availability [58,61]. In our study, E_2_ varied significantly between months. Thus, the highest values were registered in May, observing a progressive decrease in the other months, the minimum being in August. T also varied significantly between months. As in the case of E_2_, significant differences were found between the values of T in May and those of the other months, when the lowest T values were recorded. These changes are considered normal, because rainbow trout spawning takes place in spring, beginning in March and ending in April in Romania, due to the temperate-continental climate with Mediterranean influences. 

A previous study has demonstrated that E_2_ and T have opposed effects on stress responsiveness in rainbow trout, with E_2_ enhancing and T suppressing the F response to a stressor [62]. In our study, the levels of E_2_ and T found circulating in the peripheral plasma of rainbow trout varied during the four months of the summer season. The results obtained in our study are comparable to those obtained by other authors [62,63,64,65]. Regarding T, similar findings were reported by Slater and Schreck [64], who showed that testosterone was nearly as immunosuppressive as F in chinook salmon (*Oncorhynchus tshawytscha*) when lymphocytes were incubated in vitro with the steroids. They also observed that T synergizes with F to produce a greater inhibitory effect, suggesting that the two hormones act independently on different cells. The interactions between different hormones are often complex, but in many cases, changes in plasma hormone levels correspond to changes in the immune status and welfare of fish. 

## 5. Conclusions

Hematological and biochemical parameters are valuable tools for monitoring fish health. Their normal values are still undefined for the numerous farmed fish species. The results of this study reflect new aspects of blood parameters of rainbow trout in three farms and allow a better understanding of the influence of environmental conditions on the hematological and biochemical parameters in different locations. 

Clear monthly changes in the analyzed parameters were observed for all three salmonid farms studied, with the largest variation found in the case of Strâmba and Fiad farms. In future research, it would be necessary to increase the number of parameters investigated to expand the basic knowledge on the rainbow trout species and the stressors that may influence the growth and exploitation of this species.

These results will encourage efforts to expand hematological and biochemical studies to screening programs to assess the health of salmonids in intensive aquaculture in Romania, to improve immunocompetence in farmed fish, which is essential for the sustainable development of aquaculture. 

## Figures and Tables

**Table 1:** Chemical composition of feed administered in farms, according to the product label [29].

Chemical Composition (%)	Pellet 4.5 mm
Crude protein (%)	42–44
Crude fat (%)	28–30
NFE (%)	12.5–15.5
Ash (%)	6.0–8.0
Fiber (%)	0.7–1.9
Phosphorus (%)	0.9
Gross energy (MJ)	23.9–26.9
Digestible energy (MJ)	21.6

**Table 2:** Table of loadings for the physiological parameters investigated with a principal component analysis. PC1 and PC2 are displayed, which explain together 45.97% of the total variation.

Parameter ^1^	PC1 Axis	PC2 Axis
Red blood cell count (RBC)	**0.43**	0.40
Cortisol (F)	**0.35**	−0.03
Estradiol (E_2_)	**0.25**	**−0.20**
Mean corpuscular volume (MCV)	**−0.45**	−0.03
Mean corpuscular hemoglobin (MCH)	**−0.54**	−0.17
Glutathione peroxidase (GPx)	−0.11	**0.41**
Superoxide dismutase (SOD)	−0.11	**0.43**
Hematocrit (Hct)	0.03	**0.41**
Testosterone (T)	−0.17	**0.38**
Hemoglobin (Hb)	−0.29	0.22
Progesterone (PROG)	−0.05	0.14

^1^ Parameters are arranged so that all the variables that contribute strongly to principal component 1 are listed first, followed by those that contribute strongly to principal component 2, etc., is highlighted in boldface type.

**Table 3:** Summary table of the ANOVA/Kruskal–Wallis tests performed on physiological, hormonal, and hematological variables recorded over four months (May–August 2019) in three fish farms from Romania.

Type of Comparison	Variables	Effect	DF	χ^2^ Value/F Value	*p* Value
Kruskal–Wallis test	Glutathione peroxidase (GPx)	farm	2	7.79	0.020
		month	3	150.15	<0.001
	Cortisol (F)	farm	2	19.27	<0.001
		month	3	64.86	<0.001
	Estradiol (E_2_)	farm	2	0.08	0.961
		month	3	24.62	<0.001
	Testosterone (T)	farm	2	8.33	0.016
		month	3	63.07	<0.001
	Red blood cells count (RBC)	farm	2	12.19	0.002
		month	3	66.39	<0.001
	Mean corpuscular hemoglobin (MCH)	farm	2	0.31	0.856
		month	3	62.88	<0.001
	Hematocrit (Hct)	farm	2	30.67	<0.001
		month	3	43.23	<0.001
Two-way ANOVA	Superoxide dismutase (SOD)	farm	2	0.45	0.641
		month	3	40.27	<0.001
		Interaction	6	3.83	0.001
	Mean corpuscular volume (MCV)	farm	2	3.64	0.028
		month	3	7.45	<0.001
		Interaction	6	3.85	0.001

## Data Availability

The datasets generated during and/or analysed during the current study are available from the corresponding author on reasonable request.

## Supplementary Materials

The following are available online at https://www.mdpi.com/article/10.3390/ani11061810/s1, Table S1: Correlation matrices showing Spearman rank correlation coefficient between parameters recorded during May 2019 in three rainbow trout farms from Romania, Table S2: Correlation matrices showing Spearman rank correlation coefficient between parameters recorded during June 2019 in three rainbow trout farms from Romania., Table S3: Correlation matrices showing Spearman rank correlation coefficient between parameters recorded during July 2019 in three rainbow trout farms from Romania, Table S4: Correlation matrices showing Spearman rank correlation coefficient between parameters recorded during August 2019 in three rainbow trout farms from Romania.
