# Supplementary material for: Multi-Approach Assessment for Stress Evaluation in Rainbow Trout Females, Oncorhynchus mykiss (Walbaum, 1792) from Three Different Farms during the Summer Season
Source: Animals (Basel). 2021 Jun 17;11(6):1810. doi: 10.3390/ani11061810 (PMC8234804; doi:10.3390/ani11061810)
Supplement: Supplementary file 1 [file animals-11-01810-s001.zip › animals-1237114-supplementary.pdf]

Supplementary Table S1. Correlation matrices showing Spearman rank correlation coefficient between parameters recorded during May 2019 in three rainbow trout farms from Romania. Significant correlations were marked with a box in the table. Significance codes are the following: P<0.0001 '***' P<0.001 '**' P<0.01 '*' P<0.05 '.' (Abbreviations: Hb – hemoglobin, Hct – hematocrit, RBC – red blood cell count, MCV – mean corpuscular volume, MCH – mean corpuscular hemoglobin, MCHC - mean corpuscular hemoglobin concentration, T – testosterone, E_2 – estradiol, PROG – progesterone, F – cortisol, SOD – superoxide dismutase, GPx - glutathione peroxidase, Temp – temperature, Cond – Conductivity, TDS – total dissolved solids, DO – dissolved oxygen, NO_2 – nitrites, NO_3 – nitrates, NH_3 – ammonia, bdy – body temperature)

May 2019	Hb	Hct	RBC	MCV	MCH	MCHC	T	E_2	PROG	F	SOD	GPx	pH	Temp	Resist	Cond	TDS	Salinity	DO	NO_2	NO_3	NH_3	bdy
**Hb**	1	0.55***	0.4.	0.13	0.56***	0.5	0.11	-0.15	-0.06	0.01	0.18	0.08	-0.52	-0.36	0.36	-0.36	-0.36	-0.52	0.36	-0.36	0.39	-0.52	0.05
**Hct**	0.55	1	0.45***	0.35	0.17	-0.3	0.29	-0.18	-0.34	0.04	0.1	0.03	-0.58	-0.48	0.48	-0.48	-0.48	-0.58	0.48	-0.48	0.39	-0.58	-0.02
**RBC**	0.4	0.45***	1	-0.58	-0.46***	0.1	0.44*	-0.02	0.02	0.36.	0.45	0.05	-0.34	-0.67	0.67	-0.67	-0.67	-0.34	0.67	-0.67	0.01	-0.34	0.38
**MCV**	0.13	0.35.	-0.58***	1	0.68***	-0.3	-0.24	-0.13	-0.33	-0.35.	-0.4	0.04	-0.17	0.26	-0.26	0.26	0.26	-0.17	-0.26	0.26	0.35	-0.17	-0.44
**MCH**	0.56	0.17	-0.46***	0.68	1	0.4	-0.29	-0.13	-0.09	-0.32.	-0.26	0.02	-0.2	0.25	-0.25	0.25	0.25	-0.2	-0.25	0.25	0.37	-0.2	-0.35
**MCHC**	0.53	-0.32.	0.08	-0.3	0.41.	1	-0.13	0.11	0.33	0.04	0.11	0.01	-0.01	-0.02	0.02	-0.02	-0.02	-0.01	0.02	-0.02	0	-0.01	0.14
**T**	0.11	0.29	0.44***	-0.24	-0.29	-0.1	1	-0.1	-0.09	0.55	0.24	-0.1	-0.11	-0.74	0.74	-0.74	-0.74	-0.11	0.74	-0.74	-0.3	-0.11	0.6
**E_2**	-0.15	-0.18	-0.02	-0.13	-0.13	0.1	-0.1	1	0.32	0.36.	-0.07	-0.15	0.44	0.07	-0.07	0.07	0.07	0.44	-0.07	0.07	-0.46	0.44	0.14
**PROG**	-0.06	-0.34.	0.02	-0.33	-0.09	0.3	-0.09	0.32.	1	0.18	0.07	-0.19	0.21	0.17	-0.17	0.17	0.17	0.21	-0.17	0.17	-0.14	0.21	0.09
**F**	0.01	0.04	0.36.	-0.35	-0.32.	0	0.55***	0.36.	0.18	1	0.1	-0.18	0.06	-0.54	0.54	-0.54	-0.54	0.06	0.54	-0.54	-0.38	0.06	0.52
**SOD**	0.18	0.1	0.45***	-0.4	-0.26	0.1	0.24	-0.07	0.07	0.1	1	0.34.	-0.38	-0.31	0.31	-0.31	-0.31	-0.38	0.31	-0.31	0.26	-0.38	0.04
**GPx**	0.08	0.03	0.05	0.04	0.02	0	-0.1	-0.15	-0.19	-0.18	0.34	1	-0.37	-0.05	0.05	-0.05	-0.05	-0.37	0.05	-0.05	0.4	-0.37	-0.24
**pH**	-0.52	-0.58***	-0.34.	-0.17	-0.2	-0	-0.11	0.44***	0.21	0.06	-0.38	-0.37.	1	0.5	-0.5	0.5	0.5	1	-0.5	0.5	-0.87	1	0.26
**Temp**	-0.36	-0.48***	-0.67***	0.26	0.25	-0	-0.74***	0.07	0.17	-0.54***	-0.31	-0.05	0.5	1	-1	1	1	0.5	-1	1	0	0.5	-0.58
**Resist**	0.36	0.48***	0.67***	-0.26	-0.25	0	0.74***	-0.07	-0.17	0.54***	0.31	0.05	-0.5	-1	1	-1	-1	-0.5	1	-1	0	-0.5	0.58
**Cond**	-0.36	-0.48***	-0.67***	0.26	0.25	-0	-0.74***	0.07	0.17	-0.54***	-0.31	-0.05	0.5	1	-1	1	1	0.5	-1	1	0	0.5	-0.58
**TDS**	-0.36	-0.48***	-0.67***	0.26	0.25	-0	-0.74***	0.07	0.17	-0.54***	-0.31	-0.05	0.5	1	-1	1	1	0.5	-1	1	0	0.5	-0.58
**Salinity**	-0.52	-0.58***	-0.34.	-0.17	-0.2	-0	-0.11	0.44***	0.21	0.06	-0.38	-0.37.	1	0.5	-0.5	0.5	0.5	1	-0.5	0.5	-0.87	1	0.26
**DO**	0.36	0.48***	0.67***	-0.26	-0.25	0	0.74***	-0.07	-0.17	0.54***	0.31	0.05	-0.5	-1	1	-1	-1	-0.5	1	-1	0	-0.5	0.58
**NO2**	-0.36	-0.48***	-0.67***	0.26	0.25	-0	-0.74***	0.07	0.17	-0.54***	-0.31	-0.05	0.5	1	-1	1	1	0.5	-1	1	0	0.5	-0.58
**NO3**	0.39	0.39.	0.01	0.35	0.37.	0	-0.3.	-0.46***	-0.14	-0.38.	0.26	0.4.	-0.87	0	0	0	0	-0.87	0	0	1	-0.87	-0.63
**NH3**	-0.52	-0.58***	-0.34.	-0.17	-0.2	-0	-0.11	0.44***	0.21	0.06	-0.38	-0.37.	1	0.5	-0.5	0.5	0.5	1	-0.5	0.5	-0.87	1	0.26
**bdy-temp**	0.05	-0.02	0.38.	-0.44	-0.35.	0.1	0.6***	0.14	0.09	0.52***	0.04	-0.24	0.26	-0.58	0.58	-0.58	-0.58	0.26	0.58	-0.58	-0.63	0.26	1

Supplementary Table S2. Correlation matrices showing Spearman rank correlation coefficient between parameters recorded during June 2019 in three rainbow trout farms from Romania. Significant correlations were marked with a box in the table. Significance codes are the following: P<0.0001 '***' P<0.001 '**' P<0.01 '*' P<0.05 '.' (Abbreviations: Hb – hemoglobin, Hct – hematocrit, RBC – red blood cell count, MCV – mean corpuscular volume, MCH – mean corpuscular hemoglobin, MCHC - mean corpuscular hemoglobin concentration, T – testosterone, E_2 – estradiol, PROG – progesterone, F – cortisol, SOD – superoxide dismutase, GPx - glutathione peroxidase, Temp – temperature, Cond – Conductivity, TDS – total dissolved solids, DO – dissolved oxygen, NO_2 – nitrites, NO_3 – nitrates, NH_3 – ammonia, bdy – body temperature)

June 2019	Hb	Hct	RBC	MCV	MCH	MCHC	T	E_2	PROG	F	SOD	GPx	pH	Temp	Resist	Cond	TDS	Salinity	DO	NO_2	NO_3	NH_3	bdy
**Hb**	1	0.29	-0.17	0.25	0.68***	0.77	-0.31.	-0.34.	0.07	-0.09	-0.1	-0.41*	-0.55	-0.55	0.76	-0.76	-0.76	-0.76	0.55	-0.2	-0.55	-0.2	-0.52
**Hct**	0.29	1	-0.24	0.58	0.34.	-0.29	-0.11	-0.16	-0.07	0.19	0.22	-0.28	-0.11	-0.11	0.31	-0.31	-0.31	-0.31	0.11	-0.21	-0.11	-0.21	-0.07
**RBC**	-0.17	-0.24	1	-0.91	-0.79***	0.01	0.05	-0.08	-0.11	0.27	0.09	0.25	0.35	0.35	-0.2	0.2	0.2	0.2	-0.35	-0.15	0.35	-0.15	0.3
**MCV**	0.25	0.58***	-0.91***	1	0.78***	-0.13	-0.11	-0.02	0.06	-0.15	-0.04	-0.32.	-0.37	-0.37	0.31	-0.31	-0.31	-0.31	0.37	0.06	-0.37	0.06	-0.3
**MCH**	0.68	0.34.	-0.79***	0.78	1	0.46	-0.23	-0.13	0.02	-0.24	-0.1	-0.43*	-0.56	-0.56	0.59	-0.59	-0.59	-0.59	0.56	-0.03	-0.56	-0.03	-0.5
**MCHC**	0.77	-0.29	0.01	-0.13	0.46*	1	-0.22	-0.23	0.02	-0.19	-0.14	-0.19	-0.38	-0.38	0.51	-0.51	-0.51	-0.51	0.38	-0.13	-0.38	-0.13	-0.38
**T**	-0.31	-0.11	0.05	-0.11	-0.23	-0.22	1	0.15	0.15	0.08	0.36	0.22	0.35	0.35	-0.42	0.42	0.42	0.42	-0.35	0.07	0.35	0.07	0.36
**E_2**	-0.34	-0.16	-0.08	-0.02	-0.13	-0.23	0.15	1	0.11	-0.33.	0.17	0.06	0.1	0.1	-0.42	0.42	0.42	0.42	-0.1	0.32	0.1	0.32	0.05
**PROG**	0.07	-0.07	-0.11	0.06	0.02	0.02	0.15	0.11	1	-0.09	0.07	0.12	-0.22	-0.22	-0.07	0.07	0.07	0.07	0.22	0.29	-0.22	0.29	-0.2
**F**	-0.09	0.19	0.27	-0.15	-0.24	-0.19	0.08	-0.33.	-0.09	1	0.08	0.04	0.21	0.21	-0.1	0.1	0.1	0.1	-0.21	-0.11	0.21	-0.11	0.23
**SOD**	-0.1	0.22	0.09	-0.04	-0.1	-0.14	0.36.	0.17	0.07	0.08	1	-0.06	0.51	0.51	-0.2	0.2	0.2	0.2	-0.51	-0.31	0.51	-0.31	0.48
**GPx**	-0.41	-0.28	0.25	-0.32	-0.43*	-0.19	0.22	0.06	0.12	0.04	-0.06	1	0.22	0.22	-0.6	0.6	0.6	0.6	-0.22	0.38	0.22	0.38	0.13
**pH**	-0.55	-0.11	0.35.	-0.37	-0.56***	-0.38	0.35.	0.1	-0.22	0.21	0.51	0.22	1	1	-0.5	0.5	0.5	0.5	-1	-0.5	1	-0.5	0.94
**Temp**	-0.55	-0.11	0.35.	-0.37	-0.56***	-0.38	0.35.	0.1	-0.22	0.21	0.51	0.22	1	1	-0.5	0.5	0.5	0.5	-1	-0.5	1	-0.5	0.94
**Resist**	0.76	0.31.	-0.2	0.31	0.59***	0.51	-0.42*	-0.42*	-0.07	-0.1	-0.2	-0.6***	-0.5	-0.5	1	-1	-1	-1	0.5	-0.5	-0.5	-0.5	-0.47
**Cond**	-0.76	-0.31.	0.2	-0.31	-0.59***	-0.51	0.42*	0.42*	0.07	0.1	0.2	0.6***	0.5	0.5	-1	1	1	1	-0.5	0.5	0.5	0.5	0.47
**TDS**	-0.76	-0.31.	0.2	-0.31	-0.59***	-0.51	0.42*	0.42*	0.07	0.1	0.2	0.6***	0.5	0.5	-1	1	1	1	-0.5	0.5	0.5	0.5	0.47
**Salinity**	-0.76	-0.31.	0.2	-0.31	-0.59***	-0.51	0.42*	0.42*	0.07	0.1	0.2	0.6***	0.5	0.5	-1	1	1	1	-0.5	0.5	0.5	0.5	0.47
**DO**	0.55	0.11	-0.35.	0.37	0.56***	0.38	-0.35.	-0.1	0.22	-0.21	-0.51	-0.22	-1	-1	0.5	-0.5	-0.5	-0.5	1	0.5	-1	0.5	-0.94
**NO2**	-0.2	-0.21	-0.15	0.06	-0.03	-0.13	0.07	0.32.	0.29	-0.11	-0.31	0.38*	-0.5	-0.5	-0.5	0.5	0.5	0.5	0.5	1	-0.5	1	-0.47
**NO3**	-0.55	-0.11	0.35.	-0.37	-0.56***	-0.38	0.35.	0.1	-0.22	0.21	0.51	0.22	1	1	-0.5	0.5	0.5	0.5	-1	-0.5	1	-0.5	0.94
**NH3**	-0.2	-0.21	-0.15	0.06	-0.03	-0.13	0.07	0.32.	0.29	-0.11	-0.31	0.38*	-0.5	-0.5	-0.5	0.5	0.5	0.5	0.5	1	-0.5	1	-0.47
**bdy-temp**	-0.52	-0.07	0.3.	-0.3	-0.5**	-0.38	0.36.	0.05	-0.2	0.23	0.48	0.13	0.94	0.94	-0.47	0.47	0.47	0.47	-0.94	-0.47	0.94	-0.47	1

Supplementary Table S3. Correlation matrices showing Spearman rank correlation coefficient between parameters recorded during July 2019 in three rainbow trout farms from Romania. Significant correlations were marked with a box in the table. Significance codes are the following: P<0.0001 '***' P<0.001 '**' P<0.01 '*' P<0.05 '.' (Abbreviations: Hb – hemoglobin, Hct – hematocrit, RBC – red blood cell count, MCV – mean corpuscular volume, MCH – mean corpuscular hemoglobin, MCHC - mean corpuscular hemoglobin concentration, T – testosterone, E_2 – estradiol, PROG – progesterone, F – cortisol, SOD – superoxide dismutase, GPx - glutathione peroxidase, Temp – temperature, Cond – Conductivity, TDS – total dissolved solids, DO – dissolved oxygen, NO_2 – nitrites, NO_3 – nitrates, NH_3 – ammonia, bdy – body temperature)

July 2019	Hb	Hct	RBC	MCV	MCH	MCHC	T	E_2	PROG	F	SOD	GPx	pH	Temp	Resist	Cond	TDS	Salinity	DO	NO_2	NO_3	NH_3	bdy
**Hb**	1	0.41*	0.31.	0.24	0.57***	0.18	0.34.	-0.16	0.29	0.49**	0.18	-0.3.	0.36	-0.27	-0.08	0.36	0.36	-0.11	0.36	-0.36	-0.36	-0.27	-0.24
**Hct**	0.41	1	0.69***	0.56	-0.14	-0.72	0.57***	-0.31.	0.29	0.72***	0.14	-0.23	0.9	-0.42	-0.48	0.9	0.9	0.04	0.9	-0.9	-0.9	-0.42	-0.38
**RBC**	0.31	0.69***	1	-0.15	-0.52**	-0.53	0.32.	-0.21	0.27	0.56***	-0.17	-0.17	0.65	-0.28	-0.37	0.65	0.65	0.05	0.65	-0.65	-0.65	-0.28	-0.25
**MCV**	0.24	0.56***	-0.15	1	0.45*	-0.35	0.36.	-0.16	0.15	0.37.	0.36	-0.12	0.49	-0.22	-0.27	0.49	0.49	0.03	0.49	-0.49	-0.49	-0.22	-0.21
**MCH**	0.57	-0.14	-0.52**	0.45	1	0.56	-0.07	0.04	-0.02	-0.01	0.33	-0.04	-0.14	0.07	0.07	-0.14	-0.14	0	-0.14	0.14	0.14	0.07	0.04
**MCHC**	0.18	-0.72***	-0.53**	-0.35	0.56***	1	-0.46*	0.26	-0.21	-0.42*	0.07	0.05	-0.65	0.38	0.27	-0.65	-0.65	0.07	-0.65	0.65	0.65	0.38	0.3
**T**	0.34	0.57***	0.32.	0.36	-0.07	-0.46	1	-0.31.	0.37	0.58***	-0.01	-0.32.	0.6	-0.47	-0.13	0.6	0.6	-0.2	0.6	-0.6	-0.6	-0.47	-0.36
**E_2**	-0.16	-0.31.	-0.21	-0.16	0.04	0.26	-0.31.	1	-0.22	-0.41*	-0.04	0.07	-0.4	0.26	0.14	-0.4	-0.4	0.07	-0.4	0.4	0.4	0.26	0.1
**PROG**	0.29	0.29	0.27	0.15	-0.02	-0.21	0.37.	-0.22	1	0.39*	-0.13	-0.16	0.32	-0.63	0.32	0.32	0.32	-0.55	0.32	-0.32	-0.32	-0.63	-0.65
**F**	0.49	0.72***	0.56***	0.37	-0.01	-0.42	0.58***	-0.41*	0.39	1	0.02	-0.29	0.83	-0.58	-0.25	0.83	0.83	-0.19	0.83	-0.83	-0.83	-0.58	-0.51
**SOD**	0.18	0.14	-0.17	0.36	0.33.	0.07	-0.01	-0.04	-0.13	0.02	1	-0.01	0.09	-0.02	-0.07	0.09	0.09	0.03	0.09	-0.09	-0.09	-0.02	-0.02
**GPx**	-0.3	-0.23	-0.17	-0.12	-0.04	0.05	-0.32.	0.07	-0.16	-0.29	-0.01	1	-0.19	0.48	-0.29	-0.19	-0.19	0.45	-0.19	0.19	0.19	0.48	0.44
**pH**	0.36	0.9***	0.65***	0.49	-0.14	-0.65	0.6***	-0.4*	0.32	0.83***	0.09	-0.19	1	-0.5	-0.5	1	1	0	1	-1	-1	-0.5	-0.42
**Temp**	-0.27	-0.42*	-0.28	-0.22	0.07	0.38	-0.47*	0.26	-0.63	-0.58***	-0.02	0.48**	-0.5	1	-0.5	-0.5	-0.5	0.87	-0.5	0.5	0.5	1	0.9
**Resist**	-0.08	-0.48**	-0.37.	-0.27	0.07	0.27	-0.13	0.14	0.32	-0.25	-0.07	-0.29	-0.5	-0.5	1	-0.5	-0.5	-0.87	-0.5	0.5	0.5	-0.5	-0.48
**Cond**	0.36	0.9***	0.65***	0.49	-0.14	-0.65	0.6***	-0.4*	0.32	0.83***	0.09	-0.19	1	-0.5	-0.5	1	1	0	1	-1	-1	-0.5	-0.42
**TDS**	0.36	0.9***	0.65***	0.49	-0.14	-0.65	0.6***	-0.4*	0.32	0.83***	0.09	-0.19	1	-0.5	-0.5	1	1	0	1	-1	-1	-0.5	-0.42
**Salinity**	-0.11	0.04	0.05	0.03	0	0.07	-0.2	0.07	-0.55	-0.19	0.03	0.45*	0	0.87	-0.87	0	0	1	0	0	0	0.87	0.8
**DO**	0.36	0.9***	0.65***	0.49	-0.14	-0.65	0.6***	-0.4*	0.32	0.83***	0.09	-0.19	1	-0.5	-0.5	1	1	0	1	-1	-1	-0.5	-0.42
**NO2**	-0.36	-0.9***	-0.65***	-0.49	0.14	0.65	-0.6***	0.4*	-0.32	-0.83***	-0.09	0.19	-1	0.5	0.5	-1	-1	0	-1	1	1	0.5	0.42
**NO3**	-0.36	-0.9***	-0.65***	-0.49	0.14	0.65	-0.6***	0.4*	-0.32	-0.83***	-0.09	0.19	-1	0.5	0.5	-1	-1	0	-1	1	1	0.5	0.42
**NH3**	-0.27	-0.42*	-0.28	-0.22	0.07	0.38	-0.47*	0.26	-0.63	-0.58***	-0.02	0.48**	-0.5	1	-0.5	-0.5	-0.5	0.87	-0.5	0.5	0.5	1	0.9
**bdy-temp**	-0.24	-0.38.	-0.25	-0.21	0.04	0.3	-0.36.	0.1	-0.65	-0.51**	-0.02	0.44*	-0.42	0.9	-0.48	-0.42	-0.42	0.8	-0.42	0.42	0.42	0.9	1

Supplementary Table S4. Correlation matrices showing Spearman rank correlation coefficient between parameters recorded during August 2019 in three rainbow trout farms from Romania. Significant correlations were marked with a box in the table. Significance codes are the following: P<0.0001 '***' P<0.001 '**' P<0.01 '*' P<0.05 '.' (Abbreviations: Hb – hemoglobin, Hct – hematocrit, RBC – red blood cell count, MCV – mean corpuscular volume, MCH – mean corpuscular hemoglobin, MCHC - mean corpuscular hemoglobin concentration, T – testosterone, E_2 – estradiol, PROG – progesterone, F – cortisol, SOD – superoxide dismutase, GPx - glutathione peroxidase, Temp – temperature, Cond – Conductivity, TDS – total dissolved solids, DO – dissolved oxygen, NO_2 – nitrites, NO_3 – nitrates, NH_3 – ammonia, bdy – body temperature)

**August 2019**	**Hb**	**Hct**	**RBC**	**MCV**	**MCH**	**MCHC**	**T**	**E_2**	**PROG**	**F**	**SOD**	**GPx**	**pH**	**Temp**	**Resist**	**Cond**	**TDS**	**Salinity**	**DO**	**NO_2**	**NO_3**	**NH_3**	**bdy**
**Hb**	1	0.18	0.25	-0.07	0.51**	0.61	0.41*	0.16	0.26	0.02	0.08	-0.31.	0.39	0.3	-0.69	0.69	0.69	0.69	-0.3	-0.39	0.3	-0.39	0.15
**Hct**	0.18	1	0.2	0.52	-0.08	-0.64	0.01	0.17	0.05	-0.23	0	-0.09	0.28	-0.29	0.01	-0.01	-0.01	-0.01	0.29	-0.28	-0.3	-0.29	-0.26
**RBC**	0.25	0.2	1	-0.7	-0.65***	0.02	0.3.	0.47*	0.14	-0.12	0.13	-0.13	0.48	-0.31	-0.17	0.17	0.17	0.17	0.31	-0.48	-0.3	-0.47	-0.28
**MCV**	-0.07	0.52**	-0.7***	1	0.49**	-0.46	-0.25	-0.25	-0.03	-0.05	-0.04	0.04	-0.16	-0.01	0.17	-0.17	-0.17	-0.17	0.01	0.16	-0.02	0.14	-0.01
**MCH**	0.51	-0.08	-0.65***	0.49	1	0.5	0.03	-0.34	0.05	0.07	-0.12	-0.1	-0.18	0.59	-0.41	0.41	0.41	0.41	-0.59	0.18	0.58	0.17	0.47
**MCHC**	0.61	-0.64***	0.02	-0.46	0.5**	1	0.28	-0.06	0.14	0.17	0.03	-0.17	0.05	0.51	-0.56	0.56	0.56	0.56	-0.51	-0.05	0.52	-0.03	0.38
**T**	0.41	0.01	0.3.	-0.25	0.03	0.28	1	0.33.	-0.09	0.01	0.05	0.07	0.14	0.12	-0.25	0.25	0.25	0.25	-0.12	-0.14	0.12	-0.13	-0.03
**E_2**	0.16	0.17	0.47*	-0.25	-0.34.	-0.06	0.33.	1	-0.06	0.13	0.11	0.14	0.23	-0.39	0.16	-0.16	-0.16	-0.16	0.39	-0.23	-0.39	-0.23	-0.34
**PROG**	0.26	0.05	0.14	-0.03	0.05	0.14	-0.09	-0.06	1	0.07	0.14	-0.29	0.32	-0.13	-0.19	0.19	0.19	0.19	0.13	-0.32	-0.13	-0.32	-0.08
**F**	0.02	-0.23	-0.12	-0.05	0.07	0.17	0.01	0.13	0.07	1	-0.01	-0.04	-0.17	0.08	0.08	-0.08	-0.08	-0.08	-0.08	0.17	0.07	0.15	0.2
**SOD**	0.08	0	0.13	-0.04	-0.12	0.03	0.05	0.11	0.14	-0.01	1	0.24	0.33	-0.31	-0.03	0.03	0.03	0.03	0.31	-0.33	-0.3	-0.33	-0.42
**GPx**	-0.31	-0.09	-0.13	0.04	-0.1	-0.17	0.07	0.14	-0.29	-0.04	0.24	1	-0.59	-0.03	0.62	-0.62	-0.62	-0.62	0.03	0.59	-0.03	0.59	-0.09
**pH**	0.39	0.28	0.48**	-0.16	-0.18	0.05	0.14	0.23	0.32	-0.17	0.33	-0.59***	1	-0.5	-0.5	0.5	0.5	0.5	0.5	-1	-0.5	-1	-0.55
**Temp**	0.3	-0.29	-0.31.	-0.01	0.59***	0.51	0.12	-0.39*	-0.13	0.08	-0.31	-0.03	-0.5	1	-0.5	0.5	0.5	0.5	-1	0.5	1	0.5	0.87
**Resist**	-0.69	0.01	-0.17	0.17	-0.41*	-0.56	-0.25	0.16	-0.19	0.08	-0.03	0.62***	-0.5	-0.5	1	-1	-1	-1	0.5	0.5	-0.5	0.5	-0.32
**Cond**	0.69	-0.01	0.17	-0.17	0.41*	0.56	0.25	-0.16	0.19	-0.08	0.03	-0.62***	0.5	0.5	-1	1	1	1	-0.5	-0.5	0.5	-0.5	0.32
**TDS**	0.69	-0.01	0.17	-0.17	0.41*	0.56	0.25	-0.16	0.19	-0.08	0.03	-0.62***	0.5	0.5	-1	1	1	1	-0.5	-0.5	0.5	-0.5	0.32
**Salinity**	0.69	-0.01	0.17	-0.17	0.41*	0.56	0.25	-0.16	0.19	-0.08	0.03	-0.62***	0.5	0.5	-1	1	1	1	-0.5	-0.5	0.5	-0.5	0.32
**DO**	-0.3	0.29	0.31.	0.01	-0.59***	-0.51	-0.12	0.39*	0.13	-0.08	0.31	0.03	0.5	-1	0.5	-0.5	-0.5	-0.5	1	-0.5	-1	-0.5	-0.87
**NO2**	-0.39	-0.28	-0.48**	0.16	0.18	-0.05	-0.14	-0.23	-0.32	0.17	-0.33	0.59***	-1	0.5	0.5	-0.5	-0.5	-0.5	-0.5	1	0.5	1	0.55
**NO3**	0.3	-0.3.	-0.3.	-0.02	0.58***	0.52	0.12	-0.39*	-0.13	0.07	-0.3	-0.03	-0.5	1	-0.5	0.5	0.5	0.5	-1	0.5	1	0.5	0.86
**NH3**	-0.39	-0.29	-0.47*	0.14	0.17	-0.03	-0.13	-0.23	-0.32	0.15	-0.33	0.59***	-1	0.5	0.5	-0.5	-0.5	-0.5	-0.5	1	0.5	1	0.55
**bdy-temp**	0.15	-0.26	-0.28	-0.01	0.47*	0.38	-0.03	-0.34.	-0.08	0.2	-0.42	-0.09	-0.55	0.87	-0.32	0.32	0.32	0.32	-0.87	0.55	0.86	0.55	1
